# Bacterial Compositional Shifts of Gut Microbiomes in Patients with Rheumatoid Arthritis in Association with Disease Activity

**DOI:** 10.3390/microorganisms10091820

**Published:** 2022-09-11

**Authors:** Nagwan G. El Menofy, Mohammed Ramadan, Eman R. Abdelbary, Hatem G. Ibrahim, Adel I. Azzam, Mohamed M. Ghit, Ahmed S. Ezz, Yasser A. Gazar, Mohammed Salah

**Affiliations:** 1 Microbiology and Immunology Department, Faculty of Pharmacy (Girls), Al-Azhar University, Cairo 11884, Egypt; 2 Microbiology and Immunology Department, Faculty of Pharmacy Al-Azhar University—Assiut Branch, Assiut 71526, Egypt; 3 Department of Rheumatology and Rehabilitation, Faculty of Medicine, Al-Azhar University, Cairo 11651, Egypt; 4 Microbiology and Immunology Department, Faculty of Pharmacy, Port Said University, Port Said City 42526, Egypt

**Keywords:** gut microbiome, rheumatoid arthritis, autoimmune disease, 16S rRNA gene sequencing, DAS28, *Megasphaera*, *Adlercreutzia*

## Abstract

Background: Rheumatoid arthritis (RA) is a chronic inflammatory disabling autoimmune disorder. Little is known regarding the association between the gut microbiome and etiopathogenesis of RA. We aimed to dissect the differences in gut microbiomes associated with RA in comparison to healthy individuals and, in addition, to identify the shifts in the bacterial community in association with disease activity; Methods: In order to identify compositional shifts in gut microbiomes of RA patients, V3-V4 hypervariable regions of 16S rRNA were sequenced using Illumina MiSeq. In total, sixty stool samples were collected from 45 patients with RA besides 15 matched healthy subjects; Results: Notably, RA microbiomes were significantly associated with diverse bacterial communities compared with healthy individuals. Likewise, a direct association between bacterial diversity and disease activity was detected in RA patients (Kruskal Wallis; *p* = 0.00047). In general, genus-level analysis revealed a positive coexistence between RA and *Megasphaera*, *Adlercreutzia*, *Ruminococcus*, *Bacteroides*, *Collinsella,* and *Acidaminococcus*. Furthermore, Spearman correlation analysis significantly stratified the most dominant genera into distinct clusters that were mainly based on disease activity (*r* ≥ 0.6; *p* ≤ 0.05). The predictive metabolic profile of bacterial communities associated with RA could support the potential impact of gut microbiomes in either the development or recovery of RA; Conclusions: The overall shifts in bacterial composition at different disease statuses could confirm the cross-linking of certain genera either to causation or progression of RA.

## 1. Introduction

Autoimmune disorders are accompanied by immune system impairment. Understanding the basic mechanisms that drive disease initiation and progression has long been a major target in the profession [1]. RA is an autoimmune disease associated with many factors, including hormonal factors, environmental factors, genetic factors, and immune system interaction [2,3,4]. RA is characterized by persistent autoimmune reactions leading to inflammation and multiple joint destruction [5,6]. It targets self-antigens in the synovial fluids, cartilage, and bone [7]. Like other autoimmune diseases such as systemic lupus erythematosus and type 1 diabetes, RA predominates mainly in females [8].

The etiology of RA is complex and involves an interaction between the innate and acquired immunity leading to the production of autoantibodies directed against their own cellular structures as rheumatoid factor (RF) and anticitrullinated protein antibodies (ACPAs) [5]. These antibodies are often present in the blood before the appearance of joint inflammation [9]. These findings suggest that RA originates at mucosal sites, and gut and oral microbiota appear to be correlated with the onset of the disease [2]. In the past two decades, the development of effective biological treatment and small-molecule kinase inhibitors has significantly improved clinical outcomes. Cytokine inhibitors have been conclusively demonstrated to play a critical role for tumor necrosis factor-α (TNFα) and interleukin-6 in disease pathogenesis and possibly also for granulocyte-macrophage colony-stimulating factor [10,11]. Treatment of RA with disease-modifying antirheumatic drugs (DMARDs) plays an important role in controlling disease activity and acts as immunomodulators by interfering with various pro-inflammatory pathways leading to immune response suppression. Dysbiosis of the gut microbiota caused by RA can be partially restored with DMARDs treatment [12,13].

The gut microbiome, a group of microbes and their genetic contents from the gut, exerts a broad union of immunomodulatory and metabolic activities [14,15]. The gut microbiome has also been considered a new target for precision and personalized medicine, contributing to gut epithelial construction and maintenance of function, food digestion and metabolism, and immune system development [14,16]. Microbiome-targeted therapies aim to rehabilitate disturbed microbial ecosystems to a healthy or normal state, which can restore health or prevent illness [17,18]. Many recent studies and reviews have covered different sides of the microbiome and its fundamental role in human health, including the early life [19,20,21] but also specific diseases, such as inflammatory bowel diseases, cardiometabolic, atopic dermatitis, autoimmune hepatitis, cancer, obesity, and diabetes [22,23,24,25,26,27,28].

Comprehensive characterization of the structure and composition of the gut microbial ecosystem in humans is crucial for understanding the role of gut-associated bacterial communities in either healthy or disease conditions. Furthermore, it is necessary to provide an obvious description of the mechanisms involved in the pathogenesis of certain disorders as well as offer guidelines for developing prevention and treatment approaches [29]. For many decades, the majority of studies have been concerned with exploring the microbial inhabitants of the human gut; these aspects not only focused on their association with infection but also underestimated their stability over time and their interactions with other microbes [30,31,32].

Advances in sequencing technologies have given researchers further insight into the symbiotic relationship between the intestinal microbiome and its host [33].

Over the past decade, the advances in high throughput sequencing platforms led to a comprehensive understanding of the microbial communities that share human body space. With the employment of cultivation-independent sequencing technologies for the collection of individual genomes, recognition of gut microbiome investigations across individuals has increased the detected microorganisms which normally inhabit the human body [34]. 16S rRNA sequencing is today widely used as a tool for exploring the content of a microbial sample due to its relatively low cost and well-developed software analysis tools. This technology is capable of answering which microorganisms are present and their abundance. With decreased costs of DNA sequencing and improved bioinformatics tools, we can compare GI tract bacterial communities among individuals of all ages. Both 16S rDNA amplicon sequencing and the whole-genome sequencing approaches, in addition to numerous bioinformatics tools, are being deployed to tackle such vast amounts of microbiological sequence diversity and contribute to a more comprehensive understanding of human health, disease susceptibilities, and the pathophysiology of infectious and immune-mediated diseases [35].

## 2. Materials and Methods

### 2.1. Ethics Statement

This study was approved by the ethics committee of the Faculty of Pharmacy, Port Said University, Egypt (Reference no. D-7–2020). The study was performed following the principles of the Declaration of Helsinki. Informed consent was obtained from recruited patients before they participated in the study.

### 2.2. Study Design and Participants

This is a case-control study; participants were recruited during their regular follow-up visit to rheumatology outpatient clinics of Al-Hussien Hospital and Bab-Elsharia Hospital, Al-Azhar University, Cairo, Egypt. The diagnosis of RA was identified according to the criteria of the American College of Rheumatology [36].

This study was conducted on 45 patients with RA from March 2020 to June 2020. The inclusion criteria were patients with RA who were 18–60 years old, and all of them fulfilled the ACR/EULAR criteria 2010 [37]. There were exclusion criteria that included patients with a history of taking antibiotics for last three months, patients with inflammatory bowel disease, patients with sporadic colitis, and with a history of gastrointestinal surgical interference. All patients had a detailed medical history taken, which included their name, age, sex, alcohol, occupational, drug history, and any comorbid conditions they had. The erythrocyte sedimentation rate (ESR), complete blood count (CBC), rheumatoid factor (RF), C reactive protein (CRP), and anti-CCP, were among the investigations performed in the laboratory. For Assessment of disease severity for each patient, the joint was examined according to the swelling and tenderness to allow calculation of disease activity score (DAS28) (High disease activity 5.1 < DAS28, Medium disease activity 3.2 < DAS28 ≤ 5.1, Remission DAS28 ≤ 2.6) including proximal interphalangeal, metacarpophalangeal, wrists, elbows, shoulders, and knees [38]. The treatments used for RA patients were methotrexate, hydroxychloroquine, leflunomide, corticosteroids, and nonsteroidal anti-inflammatory drugs on demand. In addition, apparently normal healthy individuals not complaining of any rheumatic disorder were selected as a control group.

### 2.3. Sample Collection, DNA Extraction, and PCR Amplification and Sequencing of 16S rRNA Gene

Stool samples were obtained from the enrolled participants who fulfilled the inclusion criteria. Samples were processed and prepared for DNA extraction in the Microbiology research lab at the Faculty of Pharmacy (Girls) Al-Azhar University. DNA was extracted using DNeasy PowerMax Soil Kit (Qiagen, Hilden, Germany) according to the manufacturer’s instructions. The DNA concentration was determined by a Nanodrop spectrophotometer (Thermo Scientific™, Waltham, MA, USA). The V3-V4 regions of the 16S rRNA gene were amplified using the following primers with Illumina adaptor (underlined) as follows:

Forward Primer: 5′ TCGTCGGCAGCGTCAGATGTGTATAAGAGACAGCCTACGGGNGGCWGCAG 3′.

Reverse Primer: 5′GTCTCGTGGGCTCGGAGATGTGTATAAGAGACAGGACTACHVGGGTATCTAATCC′.

The molecular size and quality of the amplified products were investigated using agarose gel (1%) electrophoresis. Amplicons were purified by the Agencourt XP Ampure Beads (Beckam Coulter, Indianapolis, IN, USA). Finally, PCR amplicons of RA fecal samples and negative controls were sent to IGA Technology Services (Udine, Italy). They were sequenced using the Illumina MiSeq platform (Illumina, San Diego, CA, USA).

### 2.4. Bioinformatics Pipeline for Preprocessing and Analysis of 16S rRNA Sequences

Amplicon sequence variants (ASV) were employed as the base for the analysis and classification of 16S rRNA raw reads. For preprocessing of sequences, raw sequences were inputted to the Quantitative Insights Into Microbial Ecology 2 platform (QIIME2) [39]. As described previously, DADA2 plugged in QIIME2 was utilized for trimming and filtering out reads (median Phred quality ≥ 25, maximum of two expected errors per read = 2) and denoising of 16SrRNA reads (truncation length for forward = 270 bp and reverse reads = 210) and finally outputted a feature table of representative high-resolution ASVs [40]. Afterward, taxonomy assignment of ASVs was performed based on trained RDP’s naive Bayesian classifier at 97% sequence similarity [41] against SILVA reference sequences (V138) [42].

Microbial diversity analysis, based on both inter and intra-community features, was performed using QIIME2 scripts. Alpha diversity of gut microbiomes was measured using richness indices (observed species and Choa1) and Shannon diversity index for evenness. Differences in bacterial community composition in association with the health states and disease activity were tested using Permutational Multivariate Analysis of Variance (Prermanova, Mölndal, Sweden) (Adonis R, package Vegan) [43] based on both unweighted and weighted UniFrac distance matrices. The nonparametric Wilcoxon rank-sum test and Kruskal Wallis rank-sum test were performed to show the statistical significance of comparisons. The false discovery rate method (FDR) was applied to adjust the *p*-values of multiple comparisons [44].

To define the shifts in microbiomes that accompanied the different states of disease, DESeq2 was used to identify the differentially represented taxa due to disease activity (FDR-corrected *p*-value < 0.05) [45]. Also, the extensively used enterotyping approach was applied to the predominant genera in order to define the enterotypes in the entire dataset [46]. Correlations between taxa, biochemical profile, and disease activity were elucidated by applying Spearman correlation analysis on the abundant genera (mean relative abundance ≥ 0.26) using the R package, Hamsic (*r* ≥ ±0.6, *p* ≤ 0.05) [47]. Functional profiles of gut microbiomes were predicted using Tax4Fun2 based on the KEGG database [48]. Furthermore, the core taxa of the entire dataset were defined as the taxon that was detected in 80% of all samples. In contrast, the core microbiome of each health state and disease activity was defined as a taxon that was present in all samples of each group. Potential biomarkers (taxon or metabolic pathways) associated with health states and disease activity were inferred by applying the linear discriminant analysis (LDA) effective size (LEfSe) [49] on either ASVs or predicted metabolic pathways (LDA scores > 3.0, α = 0.05).

### 2.5. Data Availability

Raw data of 16S rRNA reads are accessible at https://www.ncbi.nlm.nih.gov/bioproject/PRJNA858836 (accessed on 14 July 2022); bio samples accession numbers (SAMN29768855: SAMN29768914).

Workflow for the training of RDP’s naive Bayesian present at https://github.com/mikerobeson/make_SILVA_db; https://uw-madison-microbiome-hub.github.io/Qiime2-Microbiome-workshop/ (accessed on 14 July 2022).

## 3. Results

### 3.1. Patient’s Characteristics

Sixty participants were included in this study (15 healthy controls and 45 patients with RA). The clinical characteristics and demography of participants are described in Table 1.

### 3.2. Preprocessing, Quality Filtering, and Analysis of 16S rRNA Sequences

A total of 7,143,360 raw sequences (average reads per sample = 119,056) were generated by Illumina MiSeq. Inputting of raw reads to Qiime2 generated 5,448,955 (76.28 % of all raw reads) high-quality ASVs (median length = 465 bp) that were obtained from merging forward and reverses reads, quality checking, removing low-quality reads, and potential chimeric sequences (823,629 sequences, 11.53% of all datasets and 391,456 sequences, 5.48% of all datasets, respectively).

### 3.3. Featured Taxonomic Profile of Gut Microbiomes Associated with RA

**Figure 1:**
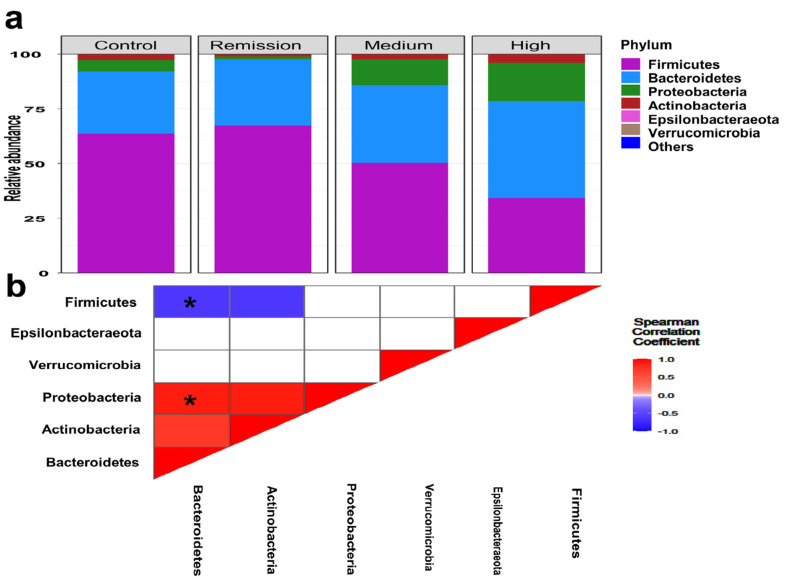
Phylum level analysis of gut microbiota. (**a**) Bar charts illustrate the relative abundance of the main phyla in the gut microbiome of studied groups. The X-axis defines the studied groups. The mean relative proportions of the dominant phyla in DAS28 groups were plotted on the Y axis. (**b**) Corrplot defines the association between the main phyla in RA microbiomes. Spearman correlation coefficient was used to define the correlation between the dominant phyla (*r* ≥ ±0.6, * *p* ≤ 0. 05).

A total of 3471 ASVs were taxonomically assigned to 17 phyla, 47 classes, 89 orders, 211 families, and 586 genera. In total, gut microbiomes associated with RA showed a remarkable representation of certain bacterial taxa at different taxonomic levels that were in contrast to healthy subjects (Figure 1a). At the phylum level, microbiomes of RA were characterized by significant enrichment of *Bacteroidetes*, *Actinobacteria,* and *Proteobacteria* over *Firmicutes* in comparison with healthy communities (Figure 1a).

On the other hand, the classification of RA samples regarding the DAS28 score revealed a significant predominance of *Firmicutes* (Mean relative abundance ± SD; 34.67 ± 13.46%, 53.42 ± 23.13% and 62.45 ± 19.79%, for high, medium, and remission, respectively). Besides, relatively variable proportions of *Proteobacteria* were detected for high, medium, and remission, respectively (Mean relative abundance SD; 2.12 ± 4.73%; 11.82 ± 6.13% and 17.39% ± 3.87%; Kruskal–Wallis, *p* = 0.00083).

Microbiomes associated with remission group were significantly accompanied by the predominance of *Firmicutes* over *Proteobacteria* and *Bacteroidetes*, in contrast to other DAS groups (Mean relative abundance of high and medium: 74.01% and 18.36%, respectively; Kruskal Wallis, *p* = 3.74 × 10^−4^). Also, significant enrichment and coexistence of *Proteobacteria* and *Bacteroidetes* (Kruskal Wallis; *p* = 3.69 × 10^−5^: Spearman; r = −0.78, *p* = 0.001) (Figure 1b).

### 3.4. Bacterial Diversity of RA Microbiomes Is Positively Linked to Disease Activity

**Figure 2:**
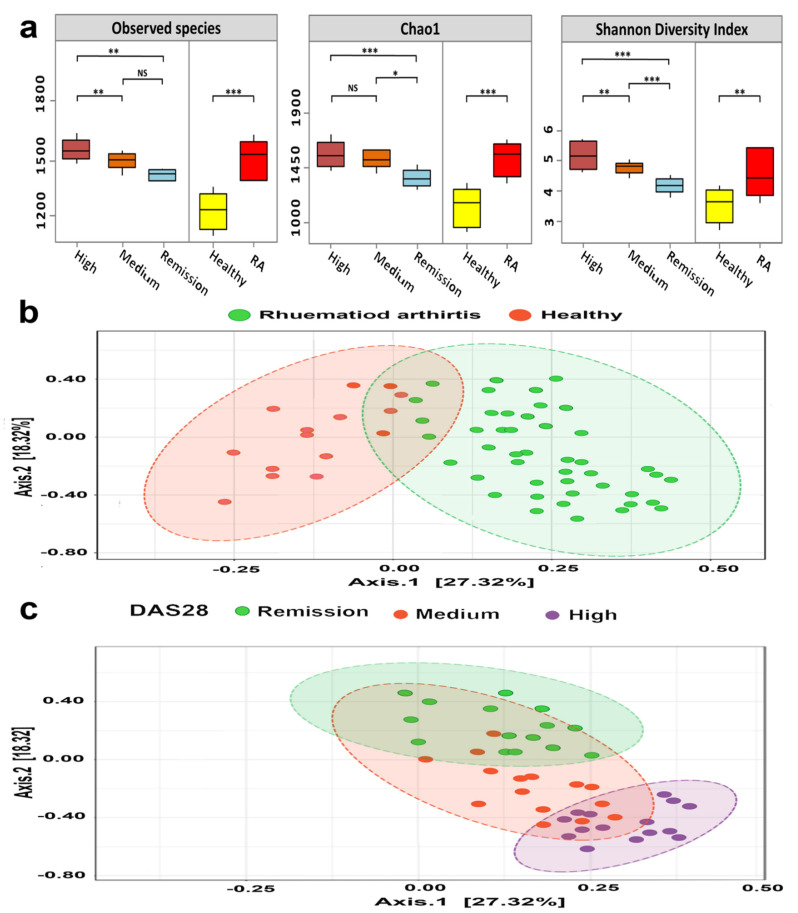
Bacterial diversity analysis of gut microbiomes. (**a**) Alpha diversity of gut microbiomes. Box plots define the bacterial diversity in terms of richness: the number of observed species and Chao1 index, and evenness; Shannon diversity index. The X-axis denotes the study groups, and the Y-axis shows the alpha diversity indices. The line in each box represents the median, the box delimits the interquartile range (IQR) between the 25th and 75th percentile, and the range was indicated by the whisker. The nonparametric Wilcoxon rank-sum test was performed to define the statistical significance of pairwise comparisons. Only significant differences were displayed with either * (*p* < 0.05), ** (*p* < 0.01) or *** (*p* < 0.001). Interindividual divergence of gut microbiomes (beta diversity) was shown in (**b**,**c**). Similarity distances between either healthy and RA microbiomes (**b**) or between RA bacterial communities (**c**) were represented by Principal coordinates analysis plots (PCoA) of gut microbiomes that were based on weighted UniFrac matrices. The significance of clustering was indicated by eclipses.

The association between the structure of microbiota and demographic characteristics of participants was investigated using both the Shannon diversity index and uniFrac matrices. Surprisingly, the gut microbiomes of RA subjects were significantly more diverse than those of healthy participants (Wilcoxon test; *p* = 2.69 × 10^−5^) (Figure 2a). Furthermore, Gut microbiomes of RA showed a markedly significant increase in bacterial diversity in association with disease activity (Kruskal–Wallis; *p* = 0.00047).

The composition and structure of RA communities showed a significant disease-based clustering (PERMANOVA; F-value: 2.8212; R-squared: 0.39433; *p* = 0.00071) (Figure 2b). Furthermore, the RA microbiomes significantly ordered to remarkable three clusters that were mainly based on disease severity (Figure 2c). On the other hand, the other studied biochemical and demographic data were contradictory to the DAS28, where there was no significant linking between the composition of gut microbiome and age, sex, ACCP, ESR, and RF (Kruskal Wallis; *p* = 0.092, 0.068, 0.63, 0.087 and 0.098, respectively).

### 3.5. The Coexistence and Differentiable Abundance of Core Genera Are Strongly Correlated to the Degree of Disease Severity

Although studying the structure of RA microbiomes at the phylum level highlighted the predominant phyla according to the health state and DAS28 as well, the underlying taxonomy levels (class, order, and family) unable to define the potential taxa that could drive the overall community in association to the healthy state.

Interestingly, Genus level analysis explained the notably diverse taxonomic profile of RA microbiomes. Regarding the health state, RA microbiomes showed the overrepresentation of *Megasphaera*, *Adlercreutzia*, *Ruminococcus*, *Bacteroides*, *Collinsella,* and *Acidaminococcus*, in contrast to those of healthy individuals. Contrarily, RA microbiomes were accompanied by diminished representation of *Acidaminococcus*, *Streptococcus*, *Gardenella*, *Anaerococcus,* and *Sphingomonas* (Supplementary Figure S1).

**Figure 3:**
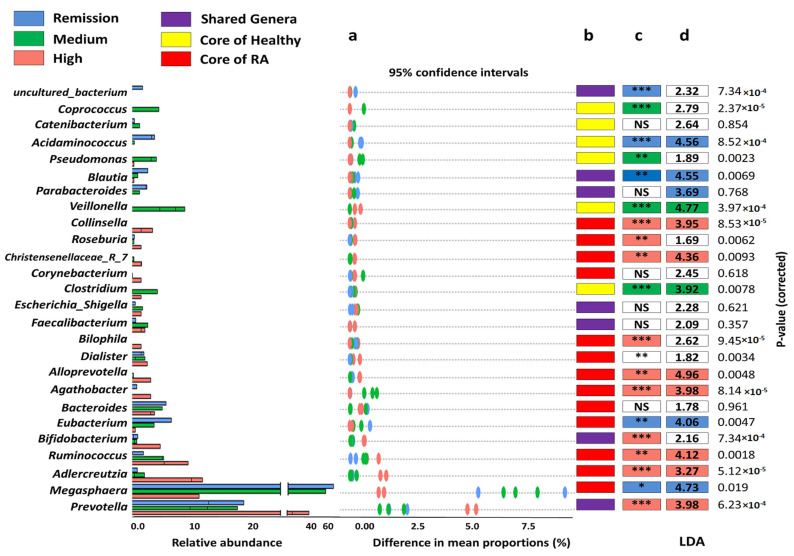
The genus level analysis of gut microbiomes in healthy and RA participants. (**a**) Bar plots define the mean proportion and differences in mean proportions with 95% confidence intervals. (**b**) The colored boxes depict the shared genera between all samples, core genera of healthy and RA gut microbiomes. (**c**) The nonparametric Wilcoxon rank-sum test was performed to define the significantly different genera between RA microbiomes based on DAS28 significant differences were displayed with either * (*p* < 0.05), ** (*p* < 0.01) or *** (*p* < 0.001). (**d**) Candidate biomarkers for each RA group were derived using LEfSe, and the numbers designate LDA scores, NS: Non-significant.

Additionally, gut microbiomes of RA provided obvious clustering that was driven by certain genera in each disease grade. The significant coexistence of *Acidaminococcus, Ruminococcus,* Christenesenelleacae_7_group *Adlercreutzia,* and *Prevotella*, markedly manifested the microbiome of high-grade DAS28. Likewise, *Adlercreutzia* and *Ruminococcus* existed with higher differential abundance in association with the high DAS28 group (Figure 3).

**Figure 4:**
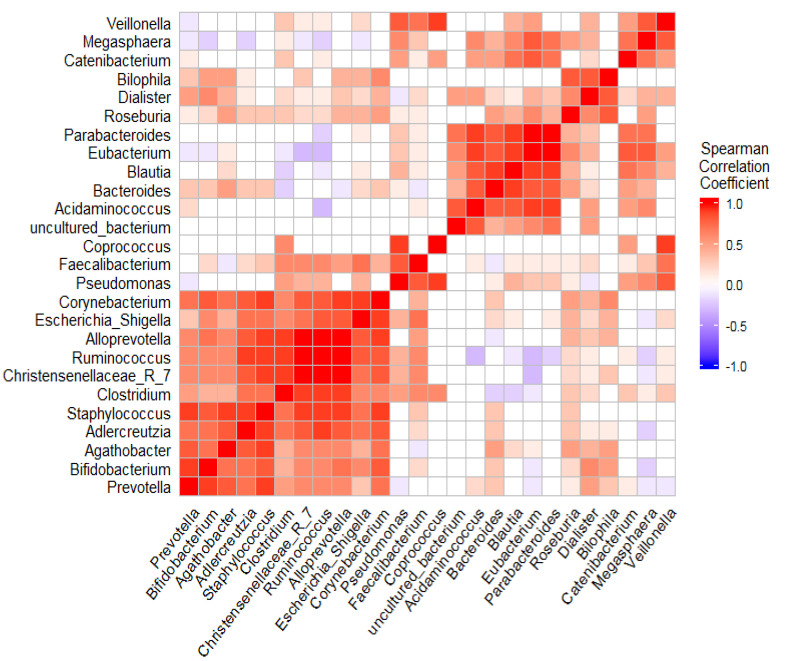
Corrplot shows the association between the most predominant genera in RA microbiomes. Spearman correlation coefficient was used to define the correlation between the dominant genera (r ≥ ±0.6, *p* ≤ 0.01).

Moreover, *Megasphaera*, *Acidaminococcus*, *Eubacterium,* and *Parabacteroides* notably had the strongest taxa correlation to the remission group (26.87, 22.65, and 21.36-fold log base 2 higher differential abundance; *p* = 2.117 × 10^−8^, 3.655 × 10^−7^ and 2.965 × 10^−7^, respectively) (Figure 4). The remarkable bacterial structure was detected with medium-grade samples. Applying the enterotyping approach to the entire dataset stratified the studied samples into five distinct enterotypes (E1–E5). The medium group was assorted to two enterotypes (E2 and E3) with a variable bacterial composition that markedly tended to either the remission group or high DAS 28 (Table 2).

### 3.6. Overall Functional Profile of Gut Microbiomes Was Likely to Contribute to the Pathogenesis of RA

The functional potential of RA gut microbiomes was inferred using Tax4Fun2. Overall, 7 and 26 metabolic pathways were detected at level 1 and Level 2, respectively. RA microbiomes were significantly associated with the upregulation of genes related to inflammatory and immune diseases such as neurodegenerative diseases, type I and II diabetes mellitus, RA, and the endocrine system. Both LEfSe and DESEq2 were employed to define the candidate biomarker and differentially abundant metabolic pathways. Microbiomes of the high DAS28 group were associated with upregulation of genes related to cell growth and death; apoptosis, bacterial motility proteins, and p53 signaling pathway, folding, sorting, and degradation such as chaperones and folding catalysts, and ubiquitin system, in addition to immune system diseases such as primary immunodeficiency. Contrarily, the overall metabolic profile of remission was significantly accompanied by an overrepresentation of membrane transport, cell motility and amino acid metabolism, carbohydrate metabolism, and ATP-binding cassette transporters.

## 4. Discussion

Since the beginning of the last decade, the journey to systematically cross-linking the human microbiome to many human diseases has been launched in Egypt [22,50,51,52]. Nevertheless, the evidence-based association about the actual role of the gut microbiome in the etiopathogenesis of rheumatoid arthritis is very limited. Several studies demonstrated the role of the gut microbiota in the pathogenesis of experimental murine arthritis. Still, the contribution of the gut microbiota in human RA has not been completely understood until recently [53,54]. Furthermore, to our knowledge, this is the first study to examine the relationship between gut microbiota and RA, specifically the degree of joint involvement.

The bacterial diversity of gut microbiomes of Chinese and Korean patients exhibited a decline in both richness and evenness [55,56,57]. Conversely, gut microbiomes of our RA patients were associated with diverse microbial communities compared with healthy individuals, which could be attributed to the well-known influence of dietary habits on species diversity of gut microbiomes, as well as geographical and social variations [58,59,60,61,62]. In addition, treatment protocols such as methotrexate significantly altered the composition and structure of gut microbiomes of RA patients that were characterized by methotrexate-tolerant bifidobacterial communities and under-representation of certain lactobacilli species [63,64]. Moreover, in accordance with previous studies, our microbiomes (healthy and diseased) showed significant disease-based clustering (Figure 2b). Also, RA microbiomes were distinctly distributed to three clusters that were based on the disease activity [55,65] as a result of perturbations in the bacterial composition of microbiomes that accompany the underlying immunological and pathophysiological of RA [56].

In agreement with previous studies, phylum level analysis of RA microbiomes was generally characterized by enrichment of *Bacteroidetes* over *Firmicutes*. On the other side, the classification of RA microbiomes in association with disease activity presented the *Firmicutes* as the dominant phyla, especially in the remission group [63,66]. The predominance of either *Bacteroidetes* or *Firmicutes* according to disease states relies on the representation of belonging genera such as *Bacteroides* or *Megasphaera*, respectively [67].

An interesting finding in the present study is the disease-associated enrichment of certain genera. *Megasphaera*, of the phylum *Firmicutes*, was previously reported with enriched abundance and positively linked to autoimmune diseases, including RA [68,69]. With respect to the predominance of *Megasphaera,* the findings of our study were in line with previous reports that support the potential role of *Megasphaera* in RA [70]. Furthermore, the low-grade disease group (remission) was significantly correlated with *Megasphaera,* that have the metabolic production abilities of essential amino acids, short chain fatty acids (SCFAs) (acetate, caproate, butyrate, and formate), and vitamins. These important products of *Megasphaera* could support its potentially beneficial healthy impact on the host [71]. Contrariwise, *Prevotella* was noticeably enriched in high-grade disease activity in contrast to medium and remission groups. This finding was consistent with the previously reported overrepresentation of *Prevotella* in pre-clinical, early, and active phases of RA, which support its pathogenic role in the pathogenesis of RA depending on its association to RA genotype, distinct gene repertoires of *Prevotella* species and carbohydrates degradation capabilities [54,58,72,73,74].

In the present study, Bacteroides was defined as a core genus in RA microbiomes, significantly correlated with RA, and were detected with variable proportions in relation to disease activity. Our finding matched with previous reports regarding the diminished abundance of *Bacteroides* at the early onset of RA followed by an increased proportion in parallel to disease severity [54,75,76,77]. The enriched abundance of *Bacteroides* could be attributed to the protective compensatory mechanisms in response to prolonged antigenic exposure in mucosa-associated lymphoid tissue [78]. Additionally, *Bacteroides* integrase antigen acts as Immunomodulator through the recruitment and proliferation of low-avidity CD8+ T cells [77]. Interestingly, tracking of relative abundance of *Megasphaera* and *Bacteroides* during treatment of RA could be a potential biomarker for the prospective prognosis.

The RA microbiomes, in comparison with healthy individuals, generally showed significant depletion of *Lactobacillus*. Lactobacilli, a known type of probiotic bacteria normally reside human intestine, perform a crucial role as immunoregulator to the host and immunomodulator for maintaining homeostasis in the gut [79,80]. This finding was consistent with a study in Shanghai that reported a significant reduction in the relative abundance of lactobacilli compared with the control group [81]. Consequently, the reduced representation of lactobacilli in RA microbiomes potentially deprives the host of its roles in maintaining intestinal homeostasis that could improve health and suppress inflammation in RA patients [82,83,84].

Of note, RA microbiomes generally showed significant enrichment of *Bifidobacterium* with variable proportions in relation to disease activity. Besides, *Bifidobacterium* was considered a core genus in a high-grade disease state and significantly overrepresented in comparison to other disease states. Intervention with *Bifidobacterium* probiotic supplements was applied to a double-blind, placebo-controlled study on the RA cohort, resulting in low disease severity. Members of *Bifidobacterium* are defined as SCFA producers [85]. SCFS, such as lactate and or acetate, could help in gut modulation in RA patients by serving as substrates for enterocytes and gut resident microbial communities [12]. Interestingly, our samples showed positive coexistence of *Bifidobacterium* and high-grade associated genera (Figure 3), which could constitute a protective compensatory response against the overwhelming predominance of *Prevotella*, *Adlercreutzia,* and *Ruminococcus* by means of gut modulation and spatial competition. *Clostridia* spp. (IV and XIVa) have also shown anti-inflammatory effects via IL-10-producing Treg cells after colonization of germ cell mice [86]. Another suggests a potential link of increased abundance of *Clostridiaceae* by tyrosine degradation pathways for inflammatory arthritis [87].

*Adlercreutzia*, *Ruminococcus*, *Collinsella,* and *Alloprevotella* were detected with relatively increased abundance in RA and positively correlated to high DAS. These findings were in line with previous reports that positively linked these genera to different inflammatory disorders. The novel finding suggests that the abundance of *Adlercreutzia* was higher in individuals with back pain, and *Adlercreutzia* abundance was positively correlated with BMI and inflammation as measured by serum leptin and adipsin concentrations. Higher *Adlercreutzia* abundance has also been correlated with lower circulating levels of non-essential amino acids, including proline and alanine, which promote bone health [88]. Gut microbiomes of patients with Spondylarthritis had an enriched abundance of *Ruminococcus gnavus*. *R. gnavus* was positively linked with the loss of the epithelial barrier that led to mucus degradation, destabilization of the intestinal barrier, and low-grade mucosal inflammation [89]. Moreover, the gut bacterium *Ruminococcus gnavus* synthesizes and secretes an inflammatory polysaccharide that induces the production of inflammatory cytokines like TNFα by dendritic cells and may contribute to the association between *R. gnavus* and Crohn’s disease [90]. Moreover, *Ruminococcus* were positively correlated with RF-IgA and anti-CCP antibodies, and *Alloprevotella* was positively correlated with numerous rheumatoid factors, such as RFIgM, RF-IgA, and RF-IgG, and with inflammatory biomarkers, including the erythrocyte sedimentation rate and C-reactive protein [81]. Chen et al. reported that the relative abundance of *Collinsella* was found to be increased in RA patients. In contrast, *Faecalibacterium*, which is generally recognized as a beneficial microbe, is decreased in RA patients. Inoculation of *Collinsella* into Collagen-induced arthritis (CIA) -susceptible mice induces severe arthritis. In vitro experiments showed that *Collinsella* increases gut permeability and induces IL-17A expression, suggesting that *Collinsella* is a candidate arthritogenic bacterium in the human intestine [53].

Particularly, the overall metabolic profile of RA microbial communities was associated with metabolic pathways that could initiate or exacerbate the disease severity. Posttranslational processes that could alter genetic events directly or indirectly can lead to the progression of RA [91]. For instance, ubiquitin was significantly enriched in the high group. Ubiquitin belongs to the family of proteins that can induce numerous diseases such as RA by reacting with Fas-induced apoptosis and rheumatoid arthritis-related synovial fibroblasts [92]. Moreover, changes in the ubiquitin proteasomal pathway led to dysregulation in cellular homeostasis that consequently resulting in RA [93]. In addition, the P53 tumor suppressor gene, as a transcription factor, has an important role in the regulation of cell division by preventing cells from uncontrolled growth. Mutation of p53 transcripts has been detected in RA joints via interfering with nuclear factor-κB (NF-κB) and mitogen-activated protein kinases (MAPKs) [93]. Lack of function of p53 by oxidative stress exerts a prominent role in the pathogenesis of RA, inflammation, immune responses, apoptosis, and cartilage degradation. Therefore, p53 may be an interesting target for RA treatment [94,95].

## 5. Conclusions

RA was collectively associated with structural and compositional shifts in the gut microbiomes as well as distinct patterns in relation to different phases of the disease. The gut microbiomes of RA potentially contributed to definite perturbations in either host-associated immunopathological factors or and normally balanced microbial ecosystem. On the other hand, members of gut microbiomes may boost the host defenses and modulate host functions toward releasing the excreted pressure of pathogenic residents, which could attenuate the induction of RA. Lastly, our findings might advocate the controversial impacts of the gut microbiome on causation as well as recovery during different disease phases.

Further study with a larger sample size for each group, in addition to studying the gut microbiomes in naïve patients in order to exclude the bidirectional influence of used medications on microbial communities, is warranted.

## Figures and Tables

**Table 1:** Summary of demographic data and clinical characteristics of the participants enrolled.

	Patients with RA (*n* = 45) *n* (%)	Control Group (*n* = 15) *n* (%)
Age (mean ± SD)	46.70 ± 12.83 years	39.46 ± 14.32 years
Sex • Male • Female	21 (46.7%) 24 (53.3%)	7 (46.7%) 8 (53.3%)
ESR (mean ± SD)	55.875 ± 29.69	NA
DAS 28 • Remission (*n* = 15) • Medium (*n* = 15) • High (*n* = 15)	2.0875 ± 0.30 4.37 ± 0.60 6.83 ± 0.77	NA
CRP (mean ± SD)	20.375 ± 20.59	NA
RF (mean ± SD)	102.125 ± 111.94	NA
ACCP (mean ± SD)	49.54 ± 105.46	NA

*n* = number of subjects, NA = not applicable, SD = standard deviation, % = percentage of all samples. The values are presented as the mean ± standard deviation or frequency. DAS28: disease activity score 28 joints, CRP: C-reactive protein, ESR: erythrocyte sedimentation rate, RF: Rheumatoid factor, ACCP: anti-cyclic citrullinated peptide.

**Table 2:** Enterotypes detected in the gut microbiome of healthy participants and RA patients.

Control	Medium	Medium	High	Remission
Enterotypes 1	Enterotypes 2	Enterotypes 3	Enterotypes 4	Enterotypes 5
*Prevotella*	*Megasphaera*	*Megasphaera*	*Prevotella*	*Megasphaera*
*Succinivibrio*	*Bacteroides*	*Prevotella*	*Adlercreutzia*	*Bacteroides*
*Faecalibacterium*	*Veillonella*	*Acidaminococcus*	*Ruminococcus*	*Agathobacter*
*Bacteroides*	*Pseudomonas*	*Eubacterium*	*Eubacterium*	*Blautia*
*Dialister*	*Faecalibazcterium*	*Bacteroides*	*Megasphaera*	*Dialister*
*Lachnospira*	*Escherichia_Shigella*	*ParaBacteroides*	*Bacteroides*	*Roseburia*
*Ruminococcus*	*Prevotella*	*Dialister*	*Agathobacter*	*Eubacterium*
*Bifidobacterium*	*ParaBacteroides*	*Ruminococcus*	*Alloprevotella*	*Bifidobacterium*
*Sutterella*	*Blautia*	*Blautia*	*Bilophila*	*ParaBacteroides*
*Streptococcus*	*Ruminococcus*	*Bifidobacterium*	*Enterobacter*	*Prevotella*
*Eubacterium*	*Dialister*		*Dorea*	
*Blautia*	*Bifidobacterium*		*Blutia*	
*Coprococcus*	*Bifidobacterium*			
*Roseburia*	*Clostridium*			
*Catenibacterium*	*Acidaminococcus*			
*Collinsella*				

## Data Availability

Available upon request.

## Supplementary Materials

The following supporting information can be downloaded at: https://www.mdpi.com/article/10.3390/microorganisms10091820/s1, Figure S1: Bar plots define the differential abundance analysis of significantly represented genera between healthy and RA individuals.
