# Supplementary figures and images for: Bacterial Compositional Shifts of Gut Microbiomes in Patients with Rheumatoid Arthritis in Association with Disease Activity
Source: Microorganisms. 2022 Sep 11;10(9):1820. doi: 10.3390/microorganisms10091820 (PMC9505928; doi:10.3390/microorganisms10091820)

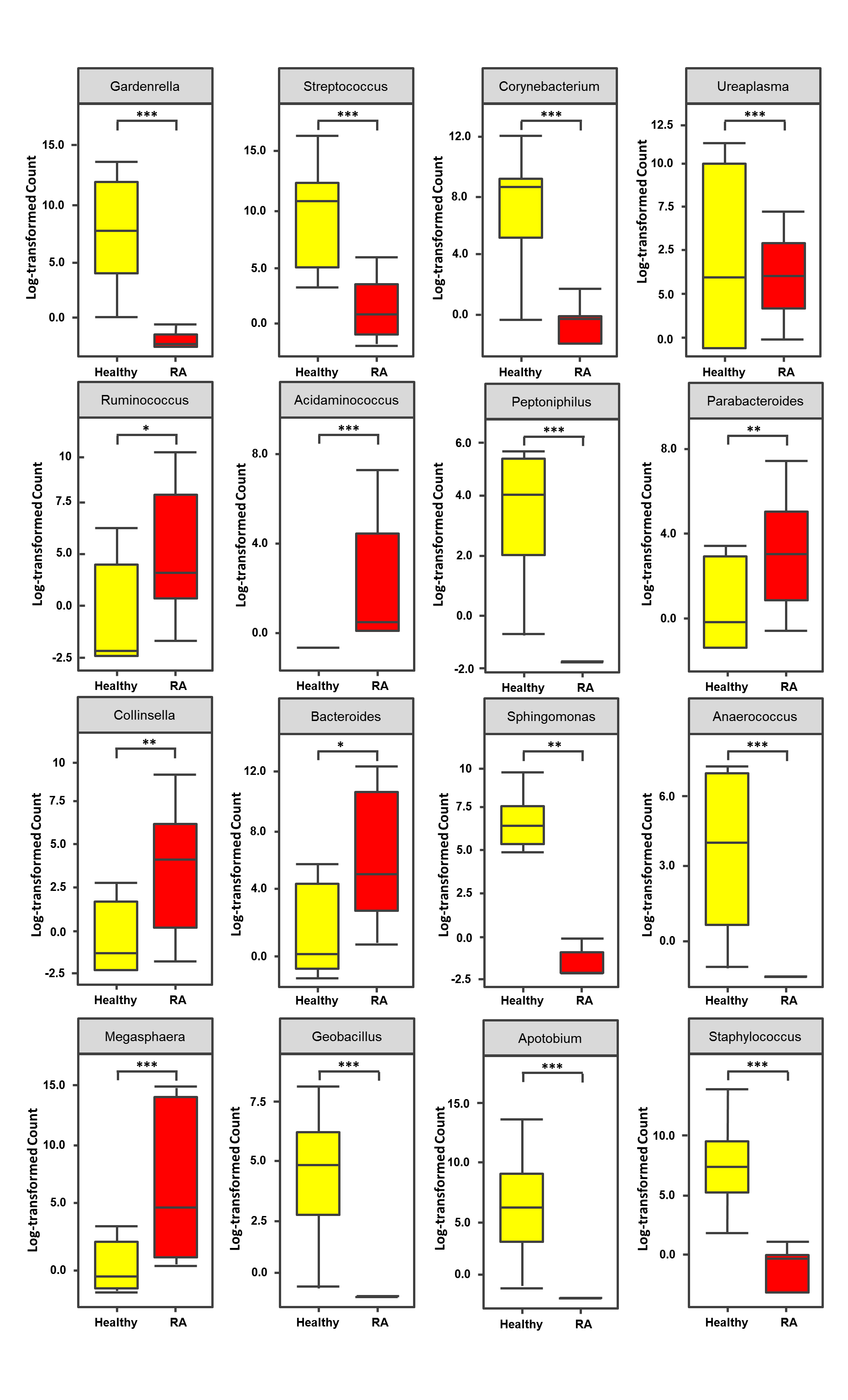

Supplement: Supplementary file 1 [file microorganisms-10-01820-s001.zip › microorganisms-1899782-supplementary.tif]
